# Clinical setting-based smoking cessation programme and the quality of life in people living with HIV in Austria and Germany

**DOI:** 10.1007/s11136-017-1580-y

**Published:** 2017-04-20

**Authors:** Igor Grabovac, Helmut Brath, Horst Schalk, Olaf Degen, Thomas E. Dorner

**Affiliations:** 1 Department of Social and Preventive Medicine, Centre for Public Health, Medical University of Vienna, Kinderspitalgasse 15/1, 1090 Vienna, Austria; 2 Health Centre South, Wienerbergstrasse 13, 1100 Vienna, Austria; 3 “Schalk-Pichler Group Practice”, Zimmermannplatz 1, 1090 Vienna, Austria; 4 Infectious Diseases Unit, University Clinic Hamburg-Eppendorf, Martinistrasse 52, 20246 Hamburg, Germany

**Keywords:** Global quality of life, Smoking, PLWHIV, Smoking cessation

## Abstract

**Purpose:**

To report on the global quality of life (QOL) in people living with HIV (PLWHIV) and how a smoking cessation intervention influences the changes in QOL.

**Methods:**

Participants were asked to fill out a questionnaire during visits to their HIV outpatient clinic consisting of sociodemographic information, general health data and the WHOQOL HIV-Bref. Exhaled carbon monoxide measurements were used to confirm the smoking status, based on which participants classified as smokers received a short 5 min structured intervention and were offered participation in a full smoking cessation programme consisting of five sessions. Follow-up was done 8 months after the baseline.

**Results:**

Overall 447 (mean age = 45.5) participants took part with 221 being classified as smokers. A total of 165 (74.6%) participants received a short intervention and 63 (29.4%) agreed to participate in the full program. At baseline, differences in QoL were observed, where smokers had lower QoL in domains of physical (*M* = 16.1 vs. 15.3, *p* = 0.009) and psychological (*M* = 15.3 vs. 14.6, *p* = 0.021) well-being, independency level (*M* = 16.1 vs. 15.2, *p* = 0.003) and environment (*M* = 16.5 vs. 16.0, *p* = 0.036). At study end, 27 (12.2%) participants quit smoking; 12 (19.0%) participants of the full programme and 15 (14.7%) that received the short intervention. There were no significant differences in QoL between those that continued to smoke and quitters at follow-up.

**Conclusion:**

Quality of life results may be used to better understand the underlying motivation of PLWHIV who start cessation programs. In order to reduce the high prevalence and health burden that smoking causes in PLWHIV, it is necessary to introduce effective interventions that can be used in the clinical settings.

## Background

Recent advances in antiretroviral therapy (ART) have improved the immune function and led to an increase in life expectancy of people living with HIV (PLWHIV) [1–3]. Yet, the health-related problems linked to cigarette smoking curtail these benefits with research indicating more than twice as many years of life lost in PLWHIV who smoke in comparison to the general population who smoke [4]. Smoking, thusly, still presents a major problem in the PLWHIV community with smoking rates being two to three times higher than in the general population with some studies also indicating a higher risk for nicotine dependence in this population [2, 5, 6].

Smoking in PLWHIV has been shown to have numerous ill-health effects including: lower pulmonary functions [7], higher incidence of cardiac disease [8], mental health issues [9, 10], decreased cognitive functioning [11], as well as on the overall HIV disease progression [12, 13] and health-related quality of life [14]. In a study that examined the overall mortality in HIV+ patients who were receiving ART, it was reported that smokers had twice as high overall mortality in comparison to non-smokers [15].

Studies concerned with influence of smoking on quality of life are usually focused on health-related quality of life (HRQOL; measures that are focused on health-related outcomes and functionality). In the general population, it was repeatedly shown that smoking is associated with lower HRQOL values [16, 17] with longitudinal studies focusing on the relationship of cessation and changes in HRQOL where smokers report lower HRQOL values in comparison to never-smokers and ex-smokers [18]. Similar focus on HRQOL is present in studies that investigate smoking in PLWHIV. These have shown that active smoking is associated with decreases in various aspects of HRQOL; general health perception, physical functioning, pain, energy and cognitive functioning [15, 19]. Compared to non-smokers, smokers also reported experiencing more symptoms (cough, shortness of breath, wheezing and chest pain), which are the strongest predictors of HRQOL in PLWHIV [19, 20].

There are numerous factors that influence smoking behaviour and hinder cessation in PLWHIV. These are usually interactive and include issues such as coping with stressful situations, social facilitation and self-medication to alleviate physical and emotional discomfort [21, 22]. Additionally, some studies outline the role of personal beliefs that influence smoking behaviour and cessation as some PLWHIV believe they will not live long enough to suffer from consequences of smoking and therefore are less concerned with the risks [22, 23]. Contrary to this and the high prevalence of smoking, some studies found many PLWHIV are interested in quitting smoking and have even reported numerous efforts to try to stop [5, 6].

Most studies deal with aspects of physical health in PLWHIV and its relation to HRQOL. Although physical health is an important part of QOL, it does not demonstrate the global QOL, which in addition to physical health includes social, recreational, spiritual and psychological dimensions [24]. There is a lack of studies investigating how smoking and smoking cessation influences the global QOL in PLWHIV, and to best of our ability, we were not able to find studies that investigate these issues in German speaking countries or elsewhere. The goal of the study was to examine whether a smoking cessation intervention aimed at PLWHIV who smoke would have an influence on the global quality of life.

## Participants and methods

### Participants

Participants were consecutive patients who visited one of the seven different participating outpatient HIV treatment centres in Austria and Germany. If the patient matched the study inclusion criteria (over 18 years of age, known serologically confirmed HIV diagnosis and signed consent) they were asked to participate. A total of 447 participants were included.

### Methods

In this multicentre interventional non-randomized study a total of six German and one Austrian HIV outpatient treatment centre participated, all located in urban regions. The study physicians and sites were recruited from the Austrian and German societies for HIV/AIDS outpatient centres. A prerequisite for physicians participating was finished education on smoking cessation methods by the Austrian or German Chamber of Medical Doctors.

After a consultation with the physician the patients were given a questionnaire to fill out by themselves, which took around 10 min to finish.

Smoking status was assessed with the question: “Have you smoked within the last 7 days?” [25, 26]. If the answer was positive the participants were further asked questions about frequency and quantity of cigarettes, if they had changed the smoking frequency after receiving the HIV diagnosis, if they were exposed to smoking at the workplace and if they had a desire to quit smoking. Smoking status was confirmed with the use of “Micro + Bedfont Smokerlyzer with Colour Touchscreen” to measure carbon monoxide (CO) in exhaled air. The Smokerlyser was calibrated, used and stored according to the instructions. All participants who had measured values above 6 ppm were considered smokers as this cutoff point was reported in literature as having high specificity and sensitivity [27–30].

The questionnaire consisted of two parts: general sociodemographic data and questions about current health (consisted of 13 open end and multiple choice questions) and the German translation of the World Health Organization Quality of Life HIV—Bref (WHOQOL HIV-Bref) questionnaire [31].

WHOQOL HIV-Bref is a 31-item short questionnaire based on the WHOQOL-Bref, a shorter form of the WHOQOL-100 with five additional items specific to PLWHIV. The items are divided into six domains (physical, psychological, level of independence, social relationships, environment and spirituality) and are scored on a Likert type scale (1–5, where “one” indicates low or negative perceptions and “five” high or positive ones) with some items not being scaled in a positive direction. As there is no official German translation of the WHOQOL HIV-Bref, the 26 items from the official German version of the WHOQOL-Bref were used and the additional five items pertaining to PLWHIV were translated to German using back translation [31, 32].

Eight months after baseline, participants were approached for a follow-up where the same protocol as in the baseline visit was used. All the collected data were placed in sealed envelopes and sent to the Medical University of Vienna for analysis without any personal information about the participants.

### Interventions

For the purposes of this study, two smoking cessation interventions were developed; a short intervention and a longer programme with five sessions. All the participants who responded affirmative to the question about the smoking status were to receive a short intervention by the physician. The structured motivational intervention lasted for about 5 min and was focused on increasing knowledge about the benefits of smoking cessation [33].

Patients were then offered to participate in the full smoking cessation programme that consisted of five individual sessions as suggested by the current guidelines of the Austrian Society for Pneumology. Time frame between the sessions was not predetermined in order to facilitate flexibility for the participants. The individual sessions were led by physicians who received formal training in smoking cessation methods and were individually structured, and lasted for around 30 min. They were focused on issues that were reported to have an effect in smoking cessation counselling, in particular strengthening problem solving skills and developing social skills in order to improve social support in the patients surroundings. According to the same guidelines, pharmacological support (nicotine replacement products, bupropion, varenicline) was offered to the patients and used only upon their wish [33]. A total of nine (4.0%) participants received nicotine replacement products with the frequency and formulation (patch, chewing gum, inhaler, lozenges) being individually chosen to best fit the lifestyle and preferences of the participants in order to increase the adherence. Another six (2.7%) patients received varenicline and four (1.8%) bupropion in the recommended dosages. All the patients were allowed to start with the full programme, including after they declined the offer following the short intervention at the first consultation.

### Statistical analysis

Descriptive statistics were performed for each variable. Quantitative variables are reported as mean values and standard deviations while the qualitative as frequency and percentage. Where appropriate a *t* test was used to determine differences in the mean values of two groups and for three or more groups, the one-way analysis of variance (ANOVA) was applied. All values below 0.05 were considered statistically significant and the analysis was performed using SPSS 21.0 statistical software (Chicago, IL., www.spss.com).

## Results

A total of 447 participants agreed to participate in the study. Response rate varied from different study centres and ranged from 69 to 98% making the overall response rate 92% at baseline. Eight months after the baseline, at study end, 47 participants were lost to follow-up, and data from 400 participants were gathered making the response rate 89.5% at study end. We observed no significant differences between the smokers and non-smokers in respect to sexual orientation, education, relationship status, CDC stage, anti-depressive and antiviral therapy or in history of cardiovascular disease or cancer. However, a binary multivariate analysis showed that smokers were more likely younger (*M* = 43.2, *p* < 0.001), male (88.2%, *p* = 0.042), more frequent IV-substance users (6.9%, *p* = 0.004) as well as having partners who also smoke (58.5% vs. 20.8%, *p* < 0.001) other sociodemographic and health-related information about the study participants is reported in Table 1.

**Table 1:** Sociodemographic data of the “VIR + smoke study” participants (*N* = 447), 2012

	Total	Smokers	Non-smokers	p*
*N*	447	221	222	
Age (years, mean value) Range: 21–82 years	45.5	43.2	47.8	<0.001
Sex (%)				0.04
Male	86.4	88.2	84.2	2
Female	11.2	8.1	14.4	
Missing	2.5	3.6	1.4	
Sexual orientation (%)				0.445
Homosexual	66.2	69.7	62.6	
Bisexual	6.7	5.4	26.1	
Heterosexual	23.9	22.2	7.7	
Missing	3.1	2.7	3.6	
Educational level (%)				0.096
Primary	13.2	15.4	11.3	
Secondary	56.4	59.3	54.5	
Tertiary	25.5	19.9	29.7	
Missing	4.9	5.4	4.5	
Relationship status (%)				0.062
In a relationship	56.0	55.7	56.3	
Not in a relationship	36.8	39.8	33.8	
Missing	7.2	4.5	9.9	
CDC stage (%)				0.627
A	85.3	56.6	56.8	
B	22.6	24.9	20.3	
C	19.0	17.2	21.2	
Missing	1.6	1.4	1.8	
Antiretroviral therapy (Yes, %)	85.3	82.2	88.0	0.104
Antidepressant therapy (Yes, %)	16.0	14.0	18.0	0.321
IV-drug use (Yes, %)	4.1	6.9	1.4	0.004
Cardiovascular disease (Yes, %)	12.4	10.2	14.7	0.161
Tumours (Yes, %)	3.8	3.2	4.6	0.470
Smoking status of the chosen physician (Yes, %)				0.478
Physician smokes	6.5	7.7	5.4	
Physician does not smoke	36.0	37.6	34.2	
Do not know	55.0	52.0	58.6	
Missing	2.5	2.7	1.8	
Smoking status of the partner^a^ (Yes, %)				<0.001
Smokes daily	39.4	58.5	20.8	
Smokes sometimes	14.3	16.3	12.0	
Does not smoke	45.4	24.4	67.2	
Missing	0.8	0.8	0.0	
Number of cigarettes smoked daily (%)				n.a.
Up to 10		24.0		
11–20		37.1		
21–30		22.2		
31 and more		13.1		
Missing		3.6		

^a^Only those participants who indicated that they have a partner

^*^Calculated using the *t*-test for data reported as mean and the *χ* ^2^ test for frequencies

Two hundred and twenty-one (49.4%) participants were classified as smokers, having reported smoking in the last 7 days and 222 as non-smokers (49.7%). Out of those who were classified as smokers, 193 (87.3%) reported to smoke daily and 82 (37.1%) consumed 11–20 cigarettes a day. One hundred and fifty-one (68.3%) reported not changing the smoking habits after the HIV diagnosis, and 124 (56.1%) said they would like to quit smoking. Measured CO values at baseline were found higher in smokers than non-smokers (16.9 vs. 4.5 ppm, *p* < 0.001) with the mean CO value for the sample was 11.2 (SD 10.5) ppm with a range from 0 to 55 ppm.

Table 2 shows the differences in baseline values of QOL stratified by number of cigarettes smoked per day. Significant differences between groups were observed for physical and psychological well-being as well as level of independence. In these domains, the mean values of QOL decreased as the daily consumption of cigarettes increased. Overall, at baseline there was a significant difference in QOL values observed between smokers and non-smokers, where smokers reported lower values in the physical and psychological well-being, level of independence and environment domains, whereas at study end there were no observed differences in mean QOL values between smokers and non-smokers as seen in Table 3.

**Table 2:** One-way analysis of variance of mean values for different domains of QoL stratified by smoking status at baseline (*N* = 432), “VIR + smoke study” 2012

Domain	*N*	Mean	SD	CI	*p*
Physical well-being					0.001
Non-smoker	216	16.07	2.68	15.71–16.43	
Up to 10	53	15.58	2.83	14.80–16.36	
11–20	84	15.79	2.66	15.21–16.37	
21–30	50	15.36	3.71	14.30–16.41	
More than 31	29	13.75	2.92	12.63–14.86	
Psychological well-being					0.002
Non-smoker	216	15.29	2.71	14.93–15.66	
Up to 10	53	14.92	3.27	14.01–15.82	
11–20	84	15.16	2.29	14.66–15.66	
21–30	50	14.24	3.01	13.38–15.10	
More than 31	29	13.26	2.99	12.12–14.40	
Level of independence					0.002
Non-smoker	216	16.10	2.93	15.71–16.49	
Up to 10	53	15.81	3.81	14.75–16.86	
11–20	84	15.67	2.79	15.06–16.27	
21–30	50	14.62	3.50	13.62–15.61	
More than 31	29	14.07	3.51	12.73–15.41	
Social relationships					0.486
Non-smoker	216	15.44	2.95	15.04–16.39	
Up to 10	53	15.58	2.93	14.77–16.39	
11–20	84	15.22	2.72	14.63–15.81	
21–30	50	15.11	3.72	14.05–16.17	
More than 31	29	14.43	3.73	13.01–15.85	
Environment					0.053
Non-smoker	216	16.51	2.30	16.20–16.82	
Up to 10	53	15.96	2.72	15.21–16.71	
11–20	84	16.27	1.94	15.85–16.69	
21–30	50	15.92	1.95	15.36–16.47	
More than 31	29	15.29	3.21	14.07–06.52	
Spirituality					0.580
Non-smoker	216	15.61	3.06	15.20–16.02	
Up to 10	53	15.86	3.40	14.92–16.80	
11–20	84	15.80	3.58	15.02–16.58	
21–30	50	15.26	3.47	14.27–16.25	
More than 31	29	14.78	4.09	13.22–16.33

**Table 3:** Differences between smokers and non-smokers in quality of life at baseline (*N* = 442) and study end (*N* = 396), “VIR + smoke study”, 2012

Quality of life domains	Smokers baseline (*N* = 221)	Non-smokers baseline (*N* = 221)	p*	Smokers study end (*N* = 197)	Non-smokers study end (*N* = 199)	p*
Physical	15.35 (3.03)	16.06 (2.68)	0.009	15.47 (2.96)	15.78 (2.93)	0.293
Psychological	14.66 (2.89)	15.28 (2.72)	0.021	14.79 (2.82)	14.67 (3.10)	0.687
Independence	15.19 (3.42)	16.09 (2.95)	0.003	15.28 (3.66)	15.48 (3.39)	0.587
Social	15.17 (3.16)	15.46 (2.93)	0.324	15.48 (3.11)	15.19 (3.27)	0.367
Environment	16.01 (2.34)	16.48 (2.32)	0.036	16.12 (2.36)	15.86 (2.77)	0.330
Spirituality	15.58 (3.56)	15.64 (3.07)	0.854	15.47 (3.41)	15.08 (3.51)	0.261

^*^ Calculated using *t*-test

According to the paired samples *t*-test (Table 4), there were no significant changes in QOL of smokers between baseline and study end. Likewise, we found no significant changes in QOL for participants who quit smoking at study end in comparison to their reported results at baseline. Interestingly, the only group that had a difference in QOL were the non-smokers, who showed a decline in mean values in psychological well-being, level of independence, environment and spirituality domains of WHOQOL HIV-Bref as presented in Table 4.

**Table 4:** Paired samples *t*-test shows changes between smokers, non-smokers and quitters in quality of life domains at between baseline (*N* = 442) and study end (*N* = 396), “VIR + smoke study”, 2012

Quality of life domains	Smokers			Non-smokers			Quitters		
	Base line	Study end	p*	Base line	Study end	p*	Base line	Study end	p*
Physical	15.42 (2.92)	15.47 (2.96)	0.778	16.09 (2.67)	15.80 (2.92)	0.052	16.03 (2.47)	15.59 (2.76)	0.459
Psychological	14.75 (2.77)	14.79 (2.83)	0.816	15.31 (2.65)	14.68 (3.10)	<0.001	15.48 (3.01)	14.99 (2.81)	0.403
Independence	15.34 (3.45)	15.38 (3.67)	0.804	16.08 (2.92)	15.49 (3.39)	0.001	15.96 (4.12)	15.33 (3.68)	0.443
Social	15.17 (3.19)	15.48 (3.11)	0.093	15.44 (2.82)	15.21 (3.27)	0.230	15.05 (3.84)	15.30 (3.28)	0.587
Environment	16.08 (2.32)	16.12 (2.36)	0.794	16.56 (2.21)	15.87 (2.78)	<0.001	16.91 (2.20)	16.67 (2.29)	0.616
Spirituality	15.61 (3.61)	15.47 (3.41)	0.538	15.65 (3.12)	15.09 (3.52)	0.010	15.47 (3.33)	14.44 (3.67)	0.134

^*^ Calculated using the paired samples *t*-test

In total 165 (76.0%) of smokers received the short intervention and 63 (28.5%) agreed to participate in the full programme. However, only 30 (47.6%) participants finished the full five sessions, while 15 (23.8%) had one, 8 (12.7%) two, 7 (11.1%) three and 3 (4.8%) four sessions. At study end, 27 (12.2%) participants reported having quit smoking, out of which 12 (19.0%) had participated in the full programme and 15 (14.7%) had only had the short intervention. Two (0.9%) patients were still using nicotine replacement products and two (0.9%) varenicline. There was a decrease from baseline in measured CO values in the whole sample (11.2 vs. 9.8, *p* < 0.001) as well as in the participants who were classified as smokers at baseline (17.5 vs. 14.8, *p* < 0.001).

At study end there were no observed differences in QOL of participants who continued to smoke versus those who quit.

## Discussion

This study aimed to show the quality of life of PLWHIV who smoke and to see the possible influence of a clinic-based smoking cessation programme. Main findings indicated that there is a baseline difference in QOL values between smokers and non-smokers, whereas smokers achieve lower values. This was further emphasized when the sample was stratified based on number cigarettes smoked where again more heavy smokers achieved lower values in physical and psychological well-being and levels of independence domains (Tables 2, 3). These results are comparable with other studies that showed lower QOL for smokers than non-smokers in the general population, as well as confirmed the findings that QOL decreases with an increase in smoking frequency [34].

Our study participants were more likely male and more frequent IV-substance users when compared to non-smokers, which is supported by literature and is in line with the general population [35, 36]. Reasons for this include biological, cultural and psychosocial issues as smoking was and still is largely an accepted form of addictive behaviour and may be associated with coping strategies [37, 38]. These individual issues such as mental health and substance and alcohol abuse as well as body image issues have been identified as barriers in smoking cessation in PLWHIV [39–42] and in the general population [43]. In addition, there are strong beliefs within the smoking community that the perceived QOL will decrease with quitting which may be a significant hindrance to smoking cessation [44]. This was not confirmed by our results, as we found no changes in mean values in QOL between baseline and study end in participants who quit smoking (Table 4).

On the other hand, smoking cessation programs are not routinely offered in the health care setting especially in the HIV care as managing HIV comorbidities and complications may be seen as more important than discussing and providing smoking cessation [45]. Additionally health care workers may not feel educated enough in smoking cessation or refrain from cessation counselling due to limited time constraints [46]. A pilot study in implementation of a smoking cessation programme aimed at PLWHIV carried out in Switzerland reported only 8% of participants agreeing to an offered smoking cessation programme [47]. In our study, participants were actively offered to take part in a smoking cessation programme by trained staff, with 30% agreed to participate and about 18% finished it. Worth noting is the high proportion of those that quit smoking actually only received the short intervention which might indicate that short interventions that do not require much time or in-depth training can be used by clinicians during day to day patient consultations, which is in line with previous studies in the general population [48].

Interestingly, our results showed no difference in QOL over time for smokers, but lower values were observed for non-smokers between baseline and study end. Such results were not found in literature, and it is possible they are related to a number of societal issues that PLWHIV face such as socioeconomic issues, stigma and discrimination and lack of social support. Some studies showed that PLWHIV who smoke report more than half of their immediate social circle also smokes indicating that smoking is maintained through social ties and may present a substitute for support for PLWHIV [49, 50].

In a randomized control trial of smoking cessation programs on 1504 participants that were not HIV positive, Piper et al. [24] reported improved global QOL in those that quit smoking after one and three years as compared to those who did not. Findings from a review by Goldenberg et al. [34] showed that QOL decreases with time spent smoking. Both these outcomes are contradictory to our results, as ours show no statistical differences between smokers and non-smokers as well as between smokers and participants that quit smoking at study end which may be linked to the relative short duration of our study or relatively small sample size. Also given the chronic nature of the HIV diagnosis, it is possible that the participants did not perceive the minor health benefits that are associated with short-term smoking cessation.

Major strengths of this study include the measurement of global QOL in PLWHIV who smoke and changes after implementation of a smoking cessation programme. To the best of our knowledge, a similar study was not conducted so far. Some of the limitations include a possible overrepresentation of male participants; however, this is expected for the PLWHIV in an extramural setting in Austria and Germany. Giving socially desirable answers might lead to a possible bias as well as rather high proportion of participants with secondary and tertiary level of education, which can influence the perceived quality of life. Finally, due to a rather short time frame of only 8 months between baseline and study end, it might have been too short of time to have an effect on the QOL.

## Conclusions

Our results showed that even short interventions based on increasing knowledge about smoking cessation benefits can be used to motivate smokers to quit. Smokers may be concerned with a decline in QOL following cessation as they view this as a disruption in routines and relationships or a loss of coping strategies, however our results did not show any significant changes in QOL between quitters and smokers. Health care providers need to work to dispel such notions as there is a growing amount of evidence showing that those who quit over long term lead more satisfied lives.
